# Metabolic Profile of *Chlamydomonas reinhardtii* in Alkaline Environment and Role in Sodic Soil Improvement and Rice Seedlings in Response to Alkaline Stress

**DOI:** 10.3390/plants15182879

**Published:** 2026-09-20

**Authors:** Dan Wang, Yunqing Zhang, Lan Zhang, Yanli Xi, Chen Zhao, Xiaoyu Feng, Min Liu, Yifan Zhang, Jiajun Sun

**Affiliations:** 1 School of Public Health, Jilin Medical University, Jilin 132013, China; 2 Microalgae Resource Development and Application Research Center, Jilin Medical University, Jilin 132013, China; 3 Jilin Provincial School-Enterprise Cooperation Innovative Laboratory of Food Nutrition and Health and Functional Food Innovation, Jilin Medical University, Jilin 132013, China

**Keywords:** *Chlamydomonas reinhardtii*, alkaline stress, metabolomics, soda–saline soil improvement, rice

## Abstract

The western part of north-eastern China is mostly covered by soda soil, which has a significant impact on the growth, breeding and production of crops. The present study investigates the physiological responses and metabolic adaptation of *Chlamydomonas reinhardtii* P.A.Dangeard (*C. reinhardtii*) under different types of alkaline stress and the potential application to soda soil improvement and plants in response to alkaline stress. The soda–saline soil was treated with *C. reinhardtii* for 5 and 120 days, respectively, and the chemical properties of the soil were determined. The microalga was cultured for a period of 12 days, with and without an alkaline medium, and then the metabolic profiles of the microalgae were then analyzed by metabolomics. Results showed that *C. reinhardtii* exhibited a high level of tolerance to 40 mM NaHCO_3_, pH 8.3, and pH 10.3 with an increase in incubation time. Moreover, *C. reinhardtii* has been demonstrated to ameliorate selected physicochemical properties of soda–saline soil by reducing soil pH, electrical conductivity (EC), and the contents of CO_3_^2−^ and HCO_3_^−^ by 40–100% (*w*/*w*, wet biomass) addition for 120 cultivation days, respectively. It was demonstrated that rice seedlings were less affected by alkaline stress in the presence of *C. reinhardtii*. Furthermore, *C. reinhardtii* has the capacity to adapt to alkaline environments by regulating key pathways such as cofactor biosynthesis, glycerophospholipid metabolism, amino acid biosynthesis, purine metabolism, carbon metabolism, tropane, piperidine, and pyridine alkaloid biosynthesis. This adaptation is characterized by the accumulation of metabolites, including saccharides, amino acids and organic acids. These interact with high pH, CO_3_^2−^ and HCO_3_^−^ ions. This study elucidates that *C. reinhardtii* has the capacity to improve soda–saline soil and respond well to diverse alkaline stresses, attributable to its intricate metabolic profile.

## 1. Introduction

Soil salinization is a global problem that has a detrimental effect on agricultural production and the health of the ecological environment. Typical characteristics of soil salinization encompass elevated pH levels and nutrient deficiency, which impose significant constraints on crop growth and soil microbial activity [1]. According to the statistics of the Food and Agriculture Organization of the United Nations (FAO), there are approximately 954 million hectares of saline–alkali land worldwide, of which 99.13 million hectares are in China [2]. The west of the Songnen Plain, located in the northeast of China, is home to one of the three largest areas of soda saline–alkali soil in the world, with a total area of more than 3 million hectares, including over 43.7 percent of heavily saline land [3]. The formation of solonetz is predominantly associated with carbonate accumulation in the soil, leading to significant alkalization, which poses considerable challenges for plant survival [4]. As Eric Ruto (2021) asserts, global crop yield losses were estimated at $27.3 billion per annum, which poses a grave threat to global agricultural production [5]. Furthermore, soda saline–alkaline soils contain sodium bicarbonate (NaHCO_3_) and sodium carbonate (Na_2_CO_3_), which are the primary factors that induce salinization of the soil and intensify environmental stress. These factors induce high pH levels, which often exceed 9.2, and exacerbate the poor structure of saline–alkali soil, as well as the lack of organic matter and nutrients. This results in severe damage to plants. This damage includes ion toxicity, oxidative stress, and pH-induced cellular dysfunction [6]. Consequently, there will be a reduction in crop yield and deterioration in soil quality. Despite the considerable challenges associated with the management of saline–alkali soils, there remains significant potential for their development and utilization. The conversion of saline–alkali lands into arable land is a process that may be achieved through scientific means.

Microalgae, a group of efficient photosynthetic autotrophs, exhibit remarkable viability and unique metabolic regulation mechanisms under abiotic stress conditions. It has been demonstrated by related studies that the maintenance of cellular homeostasis in *Chlorella vulgaris* Beijerinck (*C. vulgaris*) and *C. reinhardtii* may be facilitated through complex physiological and biochemical changes when subjected to a saline–alkali environment [7,8,9]. At the metabolic level, saline–alkali stress has been shown to induce the accumulation of substantial quantities of carbohydrates and lipids as energy storage substances in microalgal cells, with a view to coping with environmental pressure and sustaining survival [10,11]. For instance, *Chlamydomonas nivalis* (F.A.Bauer) Wille has been observed to significantly enhance fatty acid accumulation through the reallocation of carbon sources in response to abiotic stress [10]. Concurrently, the antioxidant system of microalgae performs a pivotal function in managing oxidative stress triggered by saline–alkali environments [11]. Alkaline environments are frequently associated with elevated levels of reactive oxygen species (ROS), resulting in impairment of the photosynthetic system and lipid peroxidation [11,12]. In order to alleviate such damage, microalgae initiate a series of defense mechanisms, including up-regulation of antioxidant enzymes, such as glutathione S-transferase (GST) and catalase (CAT) [13]; the accumulation of zeaxanthin, which has a photoprotective function [14]; the improvement of lipid levels and lipid biosynthesis genes [15]; and the synthesis of carotenoids, antioxidants such as polyphenols, and osmotic regulators like proline [16,17,18]. Moreover, it is imperative to acknowledge the significance of adaptive regulation of photosynthesis as a pivotal mechanism through which microalgae can exhibit tolerance to saline–alkaline stress. Research has demonstrated that saline–alkali stress may damage the donor and receptor sites of photosystem II (PSII), thereby disrupting the energy flow distribution and subsequently inhibiting the carbon assimilation efficiency in plants [19]. However, microalgae have been shown to protect photosynthetic organs from damage caused by excessive light energy by regulating Stt7 kinase-dependent phosphorylation of light-harvesting complex II (LHCII), as well as inducing the expression of proteins related to cyclic electron transport (CET) [20,21,22]. On the other hand, advanced studies have reported on the underlying alkali-tolerant physiology and molecular mechanisms of microalgae, and the studies have suggested that microalgae could effectively reduce soil pH, improve soil fertility, and promote crop growth when used as biofertilizers or in combination with nitrogen-fixing bacteria [23,24]. Ahmad (2025) reported that two main molecular mechanisms underlie microalgae–bacteria interactions: nutrient exchange and signaling molecule release [25]. Specifically, microalgae could transport organic carbon to bacteria, whereas nitrogen-fixing bacteria could release nitrogen together with micronutrients, including B vitamins and iron, to microalgae [25]. Such bacteria–microalgae interactions could further promote plant growth and ameliorate saline–alkali soil [23].

For decades, *C. reinhardtii* has been used as an established model organism in various fields of biology, including eukaryotic molecular biology, photosynthesis, light receptors, cellular metabolism, microtubule assembly dynamics, and protein transport along cilia [26]. We mainly focus on the functional characteristics of *C. reinhardtii* under abiotic stress, as well as its potential applications in promoting plant growth and improving soil. Numerous studies have investigated the response characteristics of *C. reinhardtii* to neutral salt stress, and these findings have been applied in the field of biosynthesis. For instance, NaCl stress has been demonstrated to substantially elevate the level of glycerol-3-phosphate dehydrogenase (GPDH), lysophosphatidic acid acyltransferase (LPAAT), diacylglycerol acyltransferase (DAGAT) and saturated fatty acids (SFAs) of *C. reinhardtii*, rendering this microalga a valuable source for biotechnological applications [9,27]. The content of carotenoids, polyphenols and osmoprotective molecules such as proline was increased, while oleic acid, linolenic acid, alkanes, alkenes, and phytosterols were accumulated under NaCl stress, indicating the strong adaptive capacity of *C. reinhardtii* to salt stress [11]. Furthermore, the conversion of violaxanthin to zeaxanthin increased under combined salt and high light stress, suggesting that *C. reinhardtii* possesses an important photoprotective function [14]. Additionally, Ji (2018) reported that the *C. reinhardtii* transcription factor *bZIP* (CrebZIP) may play a crucial role in mediating microalgal photosynthesis and lipid accumulation in microalgae in response to salt stress [28]. In our previous work, we compared the effects of neutral salts such as NaCl, and alkaline salts such as NaHCO_3_ and Na_2_CO_3_, on microalgae, and observed significant differences between the two types of stress. Additionally, Wang (2025) demonstrated that ChbZIP1, a bZIP family transcription factor, could enhance the tolerance of *C. reinhardtii* to alkaline environments through repair of damaged photosynthesis, increased lipid accumulation, improved fatty acid unsaturation, and enhanced antioxidant enzyme activity [29]. However, the metabolic regulatory network triggered by alkaline stress, especially differential effects induced by HCO_3_^−^ and CO_3_^2−^ in sodic soils, remains poorly characterized. Moreover, there is limited experimental evidence to support their practical utility in remediating sodic soils and alleviating alkaline-stress damage in crops. To fill these knowledge gaps, further research into the metabolic mechanisms of *C. reinhardtii* under alkaline environments, as well as its function in soil improvement and plant alkaline stress tolerance, will facilitate the elucidation of the adaptive basis of microalgae to adverse environments. Accordingly, the main objective of this work was to explore the metabolic adaptation of *C. reinhardtii* under alkaline conditions and assess its potential for sodic-soil improvement as well as its protective effects on alkaline-stressed rice seedlings. Our study also aims to compare the effects of high pH and key ions (HCO_3_^−^ and CO_3_^2−^) on this microalga. We hypothesized that *C. reinhardtii* could remodel its metabolic profile to cope with high pH stress caused by HCO_3_^−^ and CO_3_^2−^, and that microalgae application would ameliorate sodic-soil properties and mitigate alkaline-stress damage in rice seedlings. It will also provide important theoretical support and potential applications for the genetic engineering of microalgae to facilitate the ecological restoration of saline–alkali lands.

## 2. Materials and Methods

### 2.1. Microalgal Cultures and Alkaline Stress Treatments

The *C. reinhardtii* used in this study was screened and purified by Beijing Soil Ark Ecological Technology Co., Ltd. (Beijing, China) from sodic alkali soil in Baicheng, China. Species identification was verified by morphological characteristics and 18S rRNA and ITS gene barcoding. The cells were maintained in flasks containing 100 mL of Tris–acetate–phosphate (TAP) medium at 25 ± 1 °C with shaking at 100 r/min under constant illumination of 6000 Lux under a 12:12 h light:dark cycle. The TAP medium was prepared based on the method described by Gorman (1965) [30]. The microalgal strain was grown in TAP medium until it reached the exponential growth phase (optical density (OD) between 0.7 and 1 at λ = 680 nm; cell density was about 1 × 10^7^ cells/mL) [30]. The cells were then collected at 5000 r/min for 5 min at 25 °C, after which they were resuspended in TAP medium under different alkaline conditions for 12 days, including 40 mM NaHCO_3_, 20 mM Na_2_CO_3_, the pH 8.3 treatment and the pH 10.3 treatment (pH groups were adjusted with NaOH to align the pH values of 40 mM NaHCO_3_ and 20 mM Na_2_CO_3_, respectively). Cultures without alkali in TAP medium were included as a control (CK).

### 2.2. Growth Performance and Chlorophyll Content Under Alkaline Stress

To assess the morphological changes, the cells were analyzed using a microscopic analysis with a Olympus CX41 microscope (Olympus Corporation, Tokyo, Japan). Microalgae growth curves were determined at OD_680_. Endpoints were measured at 0, 3, 6, 9, and 12 days using a BioTek Epoch spectrophotometer (BioTek Instruments, Inc., Winooski, VT, USA). The microalgae’s photosynthetic pigments were extracted and analyzed at the end of the experiment using the method described by Porra 1989) [11]. The pigment concentration was calculated using the following formulae:

Chlorophyll a (mg/L) = 13.95 A665 − 6.88 A649(1)

Chlorophyll b (mg/L) = 24.96 A649 − 7.32 A665 (2)

### 2.3. Study Area and Soil Sampling

**Figure 1:**
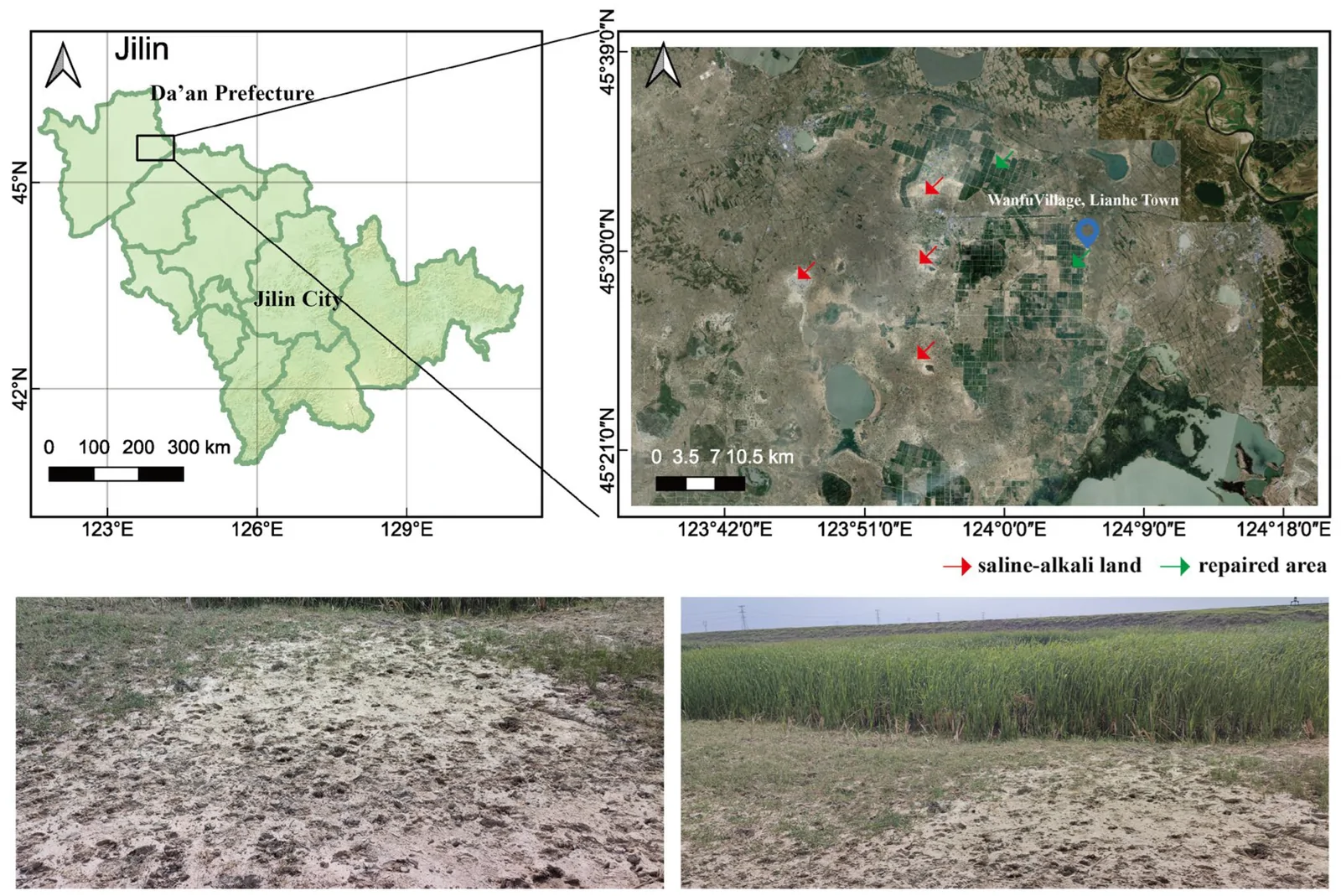
Geographic location and landform of Da’an District, Baicheng, Northeast China.

Soda saline soil was sampled on 30 June 2024 in Wanfu Village, Lianhe Town, Da’an Prefecture, Baicheng City, Jilin Province, China. This is a region that is typical of severe saline–alkali regions (124°5′24″ E, 45°30′0″ N). The geographical location and satellite images showing the landscape of saline–alkali soil in the study area are presented in Figure 1. Soil samples were taken from the 0–15 cm soil layer using a diameter auger. All samples were immediately transported to the laboratory. The soil was air-dried for physicochemical measurements. Before analysis, visible roots, stones, and coarse debris were carefully removed, after which the soil was passed through a 2 mm sieve to ensure sample homogeneity [31]. The properties of soda saline soil were determined, whose pH is 9.42, content of organic matter is 0.31 g/kg, conductivity is 367.18 μs/cm, content of carbonate is 146.78 mg/g, and content of Na^+^, K^+^, Ca^2+^, Mg^2+^, Cl^−^, SO_4_^2−^ are 0.80 mg/g, 0.02 mg/g, 0.10 mg/g, 0.06 mg/g, 0.07 mg/g, and 0.39 mg/g respectively.

### 2.4. Soil Treatment and Chemical Properties

The natural soil and the soda–saline soil were treated with *C. reinhardtii*, the concentration of which was set at concentrations of 0%, 20%, 40%, 80% and 100% (*w*/*w*, wet biomass), respectively. The 0% treatment, in which distilled water was used in place of microalgae, served as the control. During the incubation period, the level of liquid was maintained by the regular addition of the appropriate concentration of *C. reinhardtii*, whenever the liquid level fell below the soil surface. This ensured that the ratio of liquid to soil remained constant throughout the experiment.

Soil samples were collected and dried at 5 and 120 days. Then the physicochemical properties of the soil were determined, including pH, EC, HCO_3_^−^ and CO_3_^2−^. The pH and EC of the soil samples were measured using soil suspensions in a soil–water ratio of 1:5, by the potentiometric method (PHSJ-4F, Leici, Shanghai, China) and conductometry (DDSJ-308F, Leici, Shanghai, China) [32]. HCO_3_^−^ and CO^2−^ were determined by potentiometric titration methods, and three replicates were performed per treatment.

### 2.5. Plant Culture and Treatments

Rice (Jiudao-231) seeds were obtained from the Jilin Academy of Agricultural Sciences (Jilin, China). The rice seeds were germinated between moist paper towels in a light growth chamber (16 h light/8 h darkness) (GXZ-380E, Dongnan Instrument, Ningbo, China), with a light intensity of 20,000 Lux. Then, the seedlings were grown with modified Hoagland nutrient solution at 25 °C for 14 days.

Next, 14-day-old rice seedlings that reached the two-leaf and one-heart stage with a consistent average growth were selected and divided into six groups for 36 h, respectively, with one control group (CK, modified Hoagland nutrient solution without alkaline solution); NaHCO_3_ group (treated with 40 mM NaHCO_3_); Na_2_CO_3_ group (treated with 20 mM Na_2_CO_3_); microalgae group (50% *v*/*v* culture of *C. reinhardtii*); microalgae + NaHCO_3_ group (50% *v*/*v* culture of *C. reinhardtii* + 40 mM NaHCO_3_); and microalgae + Na_2_CO_3_ group (50% *v*/*v* culture of *C. reinhardtii* + 20 mM Na_2_CO_3_). The data were presented as mean values ± SD from three replicate experiments (*n* = 20 seedlings per treatment). The growth characteristics of plants were measured after treatment with alkali for 36 h.

### 2.6. Analysis of Metabolomes in Microalgae Under Alkaline Stress

The microalgae were cultured under alkaline conditions, including treatment with 40 mM NaHCO_3_ and 20 mM Na_2_CO_3_, the pH 8.3 medium and the pH 10.3 medium, respectively. The microalgae samples cultured in TAP medium without alkali supplementation served as a control group. All cultures were prepared in triplicate.

Following a 12-day cultivation period, the microalgal cells were harvested and centrifuged at 12,000 r/min for 10 min (4 °C) and washed with PBS (pH = 7.4) three times. The pellet was homogenized using a MM 400 grinder (Retsch GmbH, Haan, Germany) at a frequency of 30 Hz for 30 s. Thereafter, 50 mg of the ground sample was extracted with 600 μL of methanol/water solution (7:3, *v*/*v*) containing the internal standard. Following a thorough mixing and centrifugation process, the sample was collected and transferred for metabolomics analysis by an LC-MS system.

All samples were acquired by the LC-MS system (LC: LC-30A, Shimadzu Corporation, Kyoto, Japan; MS: TripleTOF 6600+, Sciex, Foster City, CA, USA) followed by machine orders with an ACQUITY UPLC HSS T3 1.8 µm, 2.1 mm × 100 mm column (Waters Technologies (Ireland) Ltd., Wexford, Ireland). Column temperature was set at 40 °C and the flow rate was 0.40 mL/min with a 4 μL injection volume. The mobile phase consisted of water (containing 0.1% formic acid, component A) and acetonitrile (containing 0.1% formic acid, component B). Sample measurements were performed with a gradient program that employed the starting conditions of 95% A and 5% B. Within 5 min, a linear gradient to 35% A and 65% B was programmed; within 1 min, a linear gradient to 1% A and 99% B was programmed and kept for 1.5 min. Subsequently, a composition of 95% A, 5.0% B was adjusted within 0.1 min and kept for 2.4 min. The data acquisition was performed using the information-dependent acquisition mode with Analyst TF 1.7.1 Software (Sciex Corporation, Concord, ON, Canada). Detection was performed in both positive and negative electrospray ionization (ESI) modes. The source parameters were set as follows: source temperature of 600 °C; ion spray voltage, floating, ±5500 V; and declustering potential, ±80 V. The TOF MS scan parameters were set as follows: mass range, 50–1250 Da; accumulation time, 200 ms. The product ion scan parameters were set as follows: mass range, 50–1250 Da; accumulation time, 40 ms; collision energy, ±30 or −30 V; collision energy spread, 15; resolution, UNIT; charge state, 1 to 1; intensity, 100 cps; exclude isotopes within 4 Da; mass tolerance, 50 mDa; maximum number of candidate ions to monitor per cycle, 12.

The raw MS data were converted to mzXML format using ProteoWizard MS Convert and subsequently imported into XCMS 3.20.0 (The Scripps Research Institute, La Jolla, CA, USA) for data processing.

### 2.7. Principal Component Analysis (PCA) and Hierarchical Cluster Analysis (HCA)

PCA was performed by means of the prcomp statistics function within R www.r-project.org (accessed on 21 February 2025). The data were scaled by unit variance before unsupervised PCA. The HCA results of the samples and metabolites were presented with dendrograms and carried out using the R package Complex Heatmap. For HCA, the normalized signal intensities of the metabolites (unit variance scaling) are visualized as a color spectrum. Selection of differential metabolites and Kyoto Encyclopedia of Genes and Genomes (KEGG) enrichment analysis.

In order to analyze the data relating to two groups, differential metabolites were determined using the variable importance in projection (VIP, VIP > 1) and the absolute Log2FC (|Log2FC|1.0). For multigroup analysis, differential metabolites were determined by VIP (VIP > 1) and *p*-value (*p*-value < 0.05, ANOVA). VIP values were extracted from the OPLS-DA result, which also contains score plots and permutation plots, generated using the MetaboAnalystR R package. The data were log-transformed (log_2_) and mean-centered before OPLS-DA [33].

KEGG functional enrichment analysis was performed on the differential metabolites of each group. Identified metabolites were annotated using the KEGG Compound database http://www.kegg.jp/kegg/compound/ (accessed on 21 February 2025); annotated metabolites were then mapped to the KEGG Pathway database http://www.kegg.jp/kegg/pathway.html (accessed on 21 February 2025) [34].

### 2.8. Statistical Analysis

The geographical location and the saline–alkali soil landscape of the study area were mapped using QGIS 3.44.7-Solothurn. The experiments were carried out three times, and the results are presented as the means ± standard errors. Images were obtained using GraphPad Prism 9.5.1 and Adobe Illustrator CC 2025. Data analysis was performed using IBM SPSS Statistics software version 22. A one-way analysis of variance was performed, followed by multiple-range tests with the least significant difference for different stress treatments. Volcano plots, PCA plots, circular heat maps, and Venn diagrams were performed using the Metware Cloud, a free online platform for data analysis https://cloud.metware.cn (accessed on 10 February 2026). Correlation networks between treatments and metabolites were constructed and visualized using Cytoscape v3.10.4. Pearson’s correlation coefficients were calculated in pairs with a fold change in the DPM for each treatment compared to the control. The metabolic correlation network was also constructed with Cytoscape v3.10.4. The Pearson correlation analysis was performed on metabolite abundances, and the correlation coefficient (r) > 0.9 was retained for the construction of the network.

## 3. Results

### 3.1. Microalgal Growth and Cell Morphology Under Alkaline Stress

**Figure 2:**
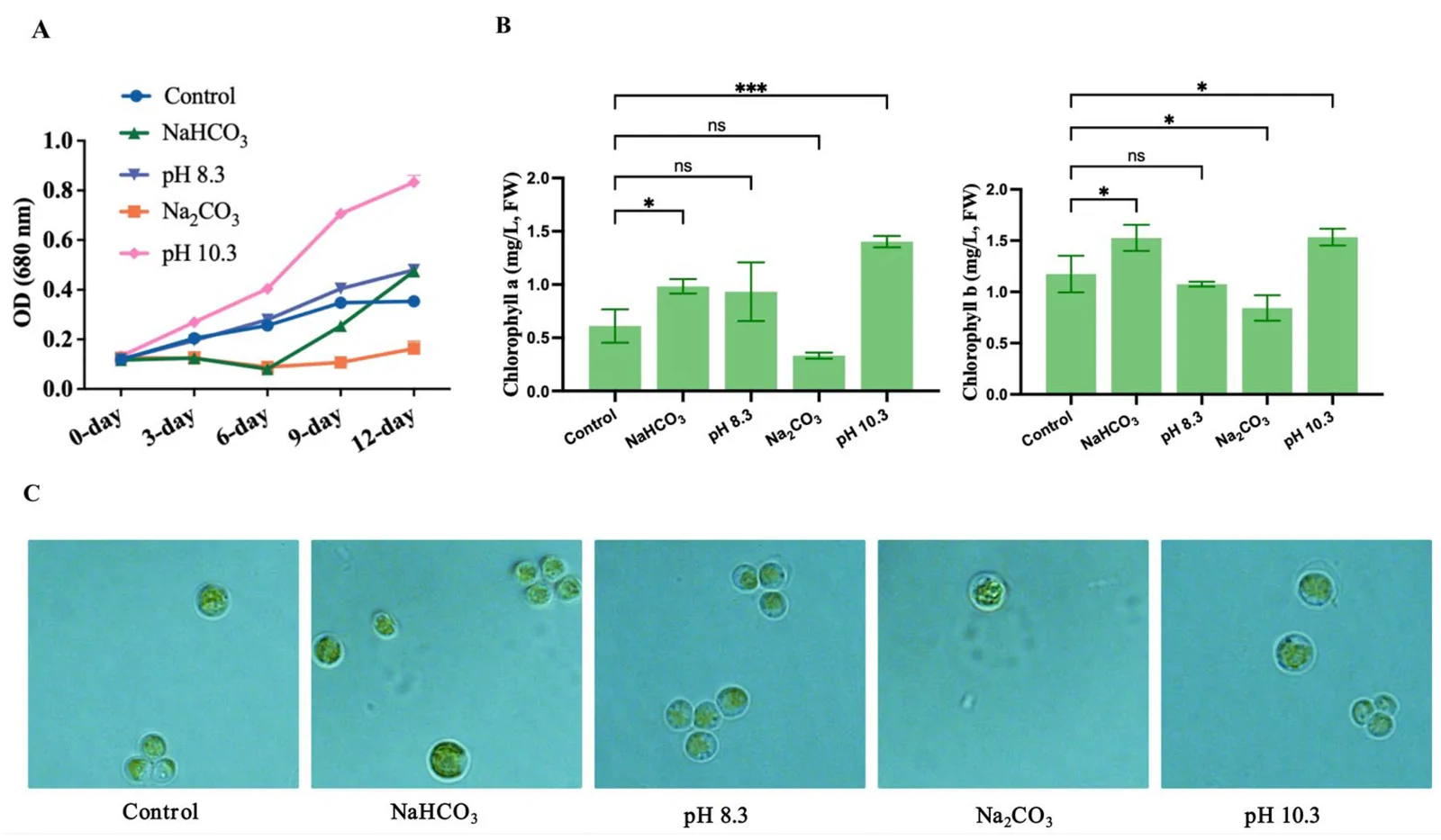
Growth and chlorophyll content of *C. reinhardtii* under different alkaline conditions. (**A**) Growth curves of *C. reinhardtii* (OD680); (**B**) chlorophyll a and chlorophyll b content of *C. reinhardtii*; (**C**) microscopic status of *C. reinhardtii*. Asterisks indicates significant differences, * *p* < 0.05, *** *p* < 0.001, and ns indicates no significant difference.

The experimental results presented in Figure 2 provide a comprehensive analysis of the responses of *C. reinhardtii* under different alkaline stresses. The growth curve based on OD_680_ (Figure 2A) quantified the growth differences in *C. reinhardtii* among the different conditions. Compared to the control group, microalgae growth was apparently inhibited under NaHCO_3_ and Na_2_CO_3_ treatments during the initial 6 days, and after which the OD exhibited an increase at a subsequent stage. In contrast, the value of the NaHCO_3_ group exhibited a marked increase, while the Na_2_CO_3_ group showed a slight increase albeit with an overall suppressed trend. However, the microalgae samples treated at pH 8.3 and pH 10.3 exhibited the most significant increase in biomass compared to the control group, especially at pH 10.3, where values exceeding 0.8 were recorded. The results demonstrated that the microalga exhibited rapid growth under NaHCO_3_ stress, pH 8.3, and pH 10.3 environments compared to the control group. This finding suggests that microalgae possess a notable degree of tolerance to specific forms of alkaline stress.

The effects of alkaline treatments on the chlorophyll content in microalgae were detected (Figure 2B). In comparison with the control group, the chlorophyll a content in the NaHCO_3_ group and the pH 10.3 group increased significantly, with the pH 10.3 group demonstrating the most substantial increase (*p* < 0.01). A comparison of the microalgae samples at pH 8.3 with the control group revealed no statistically significant differences (*p* > 0.05). Conversely, the Na_2_CO_3_ group exhibited a content of chlorophyll a that was approximately one-third of that observed in the control group. This finding suggests that the synthesis of chlorophyll was impeded by the Na_2_CO_3_ treatment. The trend of chlorophyll b content exhibited a high degree of similarity to that of chlorophyll a, with the NaHCO_3_ group and the pH 10.3 group demonstrating significantly higher levels than the control group, while the Na_2_CO_3_ group exhibited a significant decrease. The altered chlorophyll content suggests possible modifications of the photosynthetic apparatus.

The results demonstrated that certain alkaline conditions may potentially enhance photosynthesis and chlorophyll synthesis in the microalgae. However, the four alkaline environments exhibited distinct effects on *C. reinhardtii*. The chlorophyll synthesis of the pH 8.3 group did not show significant changes, while NaHCO_3_ markedly stimulated photosynthesis even at pH 8.3, indicating the dual role of NaHCO_3_, not only in terms of alkaline stress, but also HCO_3_^−^ stress, which may be another factor supporting microalgae growth. The findings of this study demonstrate that microalgae attained optimal growth and the highest chlorophyll content at a pH value of 10.3, thereby confirming their capacity to adapt to strongly alkaline conditions. Conversely, Na_2_CO_3_, which has a pH of 10.3, exhibited an inhibitory effect on photosynthesis and growth, thereby elucidating its complex role in microalgae. It can be posited that CO_3_^2−^ is the primary negative factor for the strain.

Microscopic observations revealed distinct morphological patterns of *C. reinhardtii* cells under different alkaline treatments (Figure 2C). In the control group, cells were predominantly observed to be dispersed or aggregated. The NaHCO_3_ and pH 8.3 groups exhibited a higher cell density, while the pH 10.3 group displayed a morphology similar to that of the control. The Na_2_CO_3_ group exhibited a reduced number of cells without any abnormalities, indicating that Na_2_CO_3_ impeded the proliferation of microalgae during short-term cultivation without compromising cellular structure.

The results also illustrated the high adaptability of *C. reinhardtii* to alkaline stresses. Moreover, it evinces different responses to various types of alkaline environments. The present study provides a fundamental experimental basis for the subsequent analysis of the alkali-resistant metabolic mechanism of *C. reinhardtii* and the application of microalgae in soil improvement.

### 3.2. Role of C. reinhardtii in the Improvement of Soda–Saline Soils

**Figure 3:**
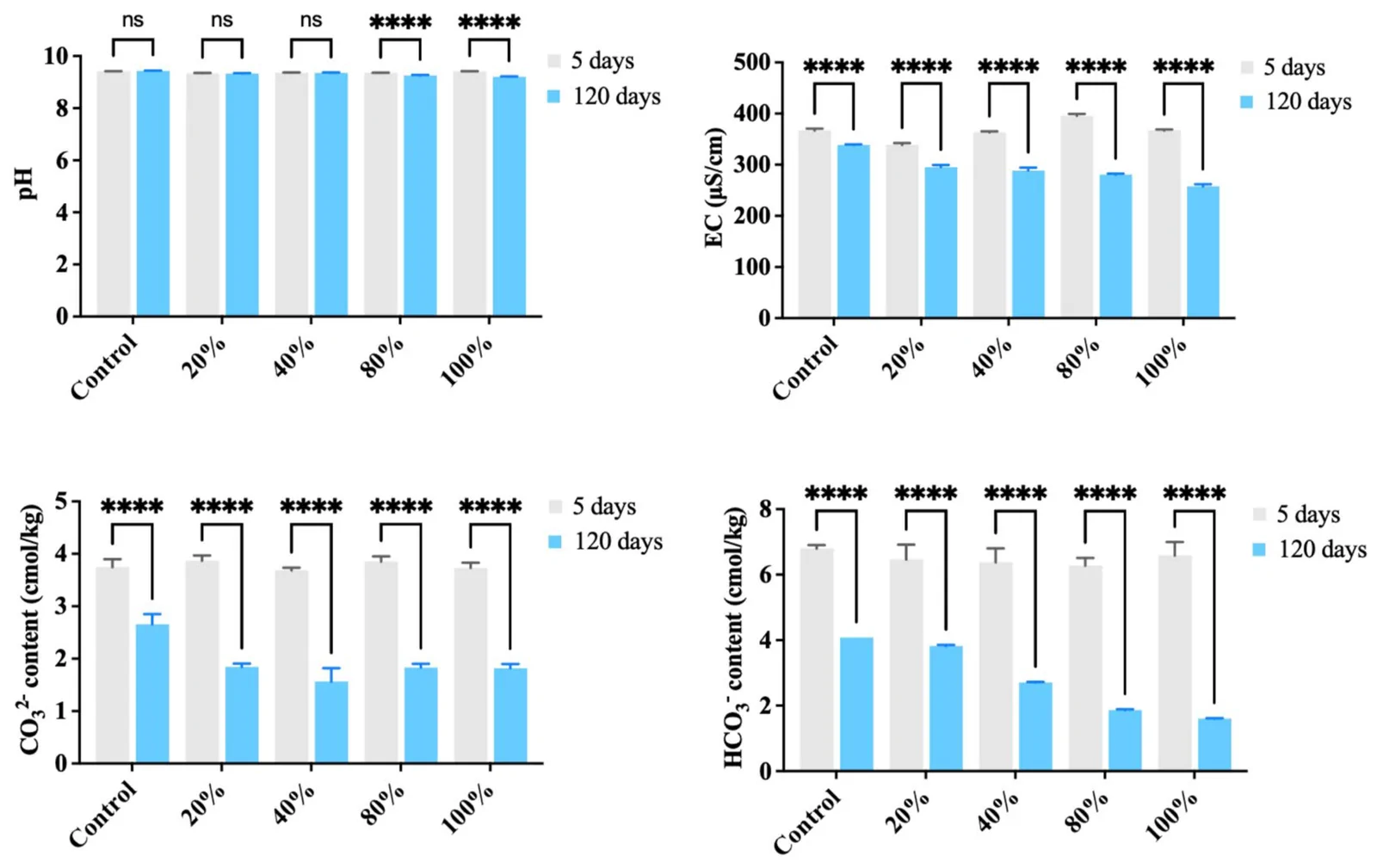
Effects of different concentrations of *C. reinhardtii* on physicochemical properties of soda−alkaline soil (5 days and 120 days). Asterisks indicates significant differences, **** *p* < 0.0001, and ns indicates no significant difference.

The remediation of soda saline soil by *C. reinhardtii* was carried out by detecting pH, EC, HCO_3_^−^, and CO_3_^2−^ content of the soil after 5 days and 120 days of treatment with *C. reinhardtii* (Figure 3). The physicochemical properties of soda saline soil treated with different concentrations of *C. reinhardtii* have been revealed, thus demonstrating the concentration and time effects of microalgae on soil improvement. In soda saline soil, a decline in pH was observed, from 9.35 to 9.26 at 80% treatment concentration, and from 9.40 to 9.20 at 100% treatment concentration. This decline was found to be statistically significant. Conversely, no substantial disparities were observed between the 5-day and 120-day soil samples at lower concentrations.

Furthermore, the EC, CO_3_^2−^, and HCO_3_^−^ levels of soda soil after the 120-day treatment showed a significant reduction compared to those after the 5-day treatment. It is noteworthy that the values of these properties underwent a gradual decline with an increasing microalgae concentration, exclusively in the 120-day-treated soda soil. In contrast, no consistent trend was observed in the 5-day−treated soil. This finding suggests that the positive effects of *C. reinhardtii* on soda soil are amplified with an extended treatment duration.

These results further support the remediation effect of *C. reinhardtii* on soda–saline soil. The process has been shown to alleviate soil alkaline stress by reducing soil pH and EC values, as well as the content of CO_3_^2−^ and HCO_3_^−^. This finding provides experimental evidence for the application of *C. reinhardtii* to the remediation of alkaline soils.

### 3.3. Role of C. reinhardtii in the Growth and Physiological Characteristics of Rice Seedlings Under Alkaline Stress

**Figure 4:**
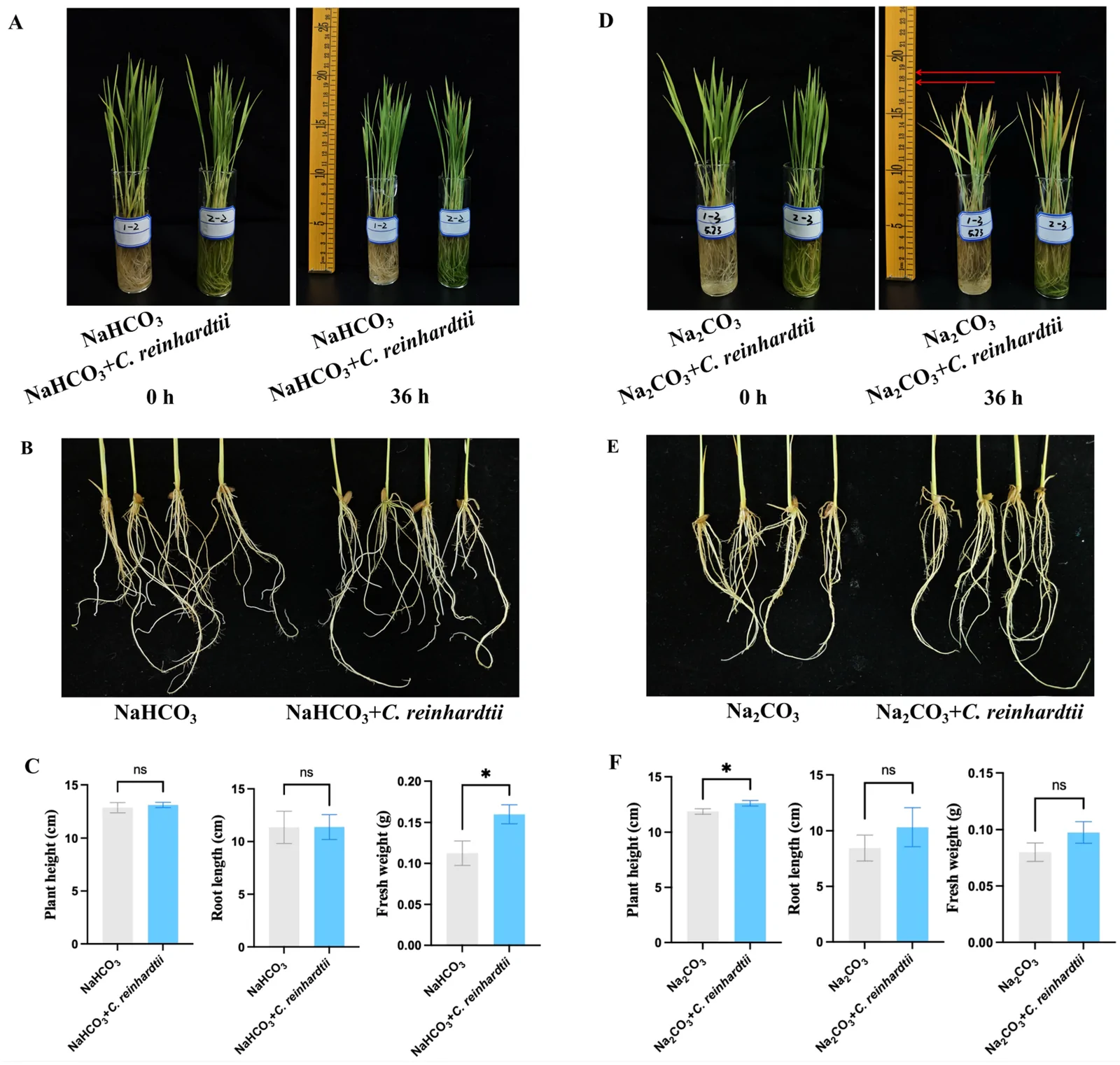
Effect of *C. reinhardtii* on the growth characteristics of rice seedlings in response to alkaline stress. Asterisks indicate significant differences in the comparison group, * *p* < 0.05, and ns indicates no significant difference. (**A**) Rice seedlings growth with or without *C. reinhardtii*−treatment under NaHCO_3_; (**B**) Rice root growth with or without *C. reinhardtii*−treatment under NaHCO_3_; (**C**) Rice seedlings growth with or without *C. reinhardtii*−treatment under Na_2_CO_3_; (**D**) Rice root growth with or without *C. reinhardtii*−treatment under Na_2_CO_3_; (**E**) Plant height, root growth and fresh weight of rice seedlings with or without *C. reinhardtii*−treatment under NaHCO_3_; (**F**) Plant height, root growth and fresh weight of rice seedlings with or without *C. reinhardtii*−treatment under Na_2_CO_3_.

Fourteen-day-old seedlings were subjected to treatment with and without microalgae under 20 mM Na_2_CO_3_ and 40 mM NaHCO_3_ for a duration of 36 h, with the objective of verifying the role of microalgae in rice seedlings in response to alkaline stress (Figure 4). In comparison with the NaHCO_3_ group, the seedlings that were exposed to microalgae demonstrated better performance. The results of the experiment demonstrated that the plant height and root length of the microalgae + NaHCO_3_ group exhibited an increase compared to the NaHCO_3_ group (*p* ˃ 0.5). However, the fresh weight of the microalgae + NaHCO_3_ group demonstrated a significant increase compared to the NaHCO_3_ group (*p* ˂ 0.5) (Figure 4A–C). In addition, microalgae were hypothesized to play a vital role in the seedlings in response to Na_2_CO_3_ stress. In comparison with the Na_2_CO_3_ group, the plant height of the microalgae group exhibited a significant increase. Furthermore, the fresh weight and root length of the microalgae group were found to be higher than those of the Na_2_CO_3_ group, though this increase was not deemed to be significant (*p* ˃ 0.5) (Figure 4D–F).

### 3.4. Microalgae Metabolism Characteristics in Response to Alkaline Stress

**Figure 5:**
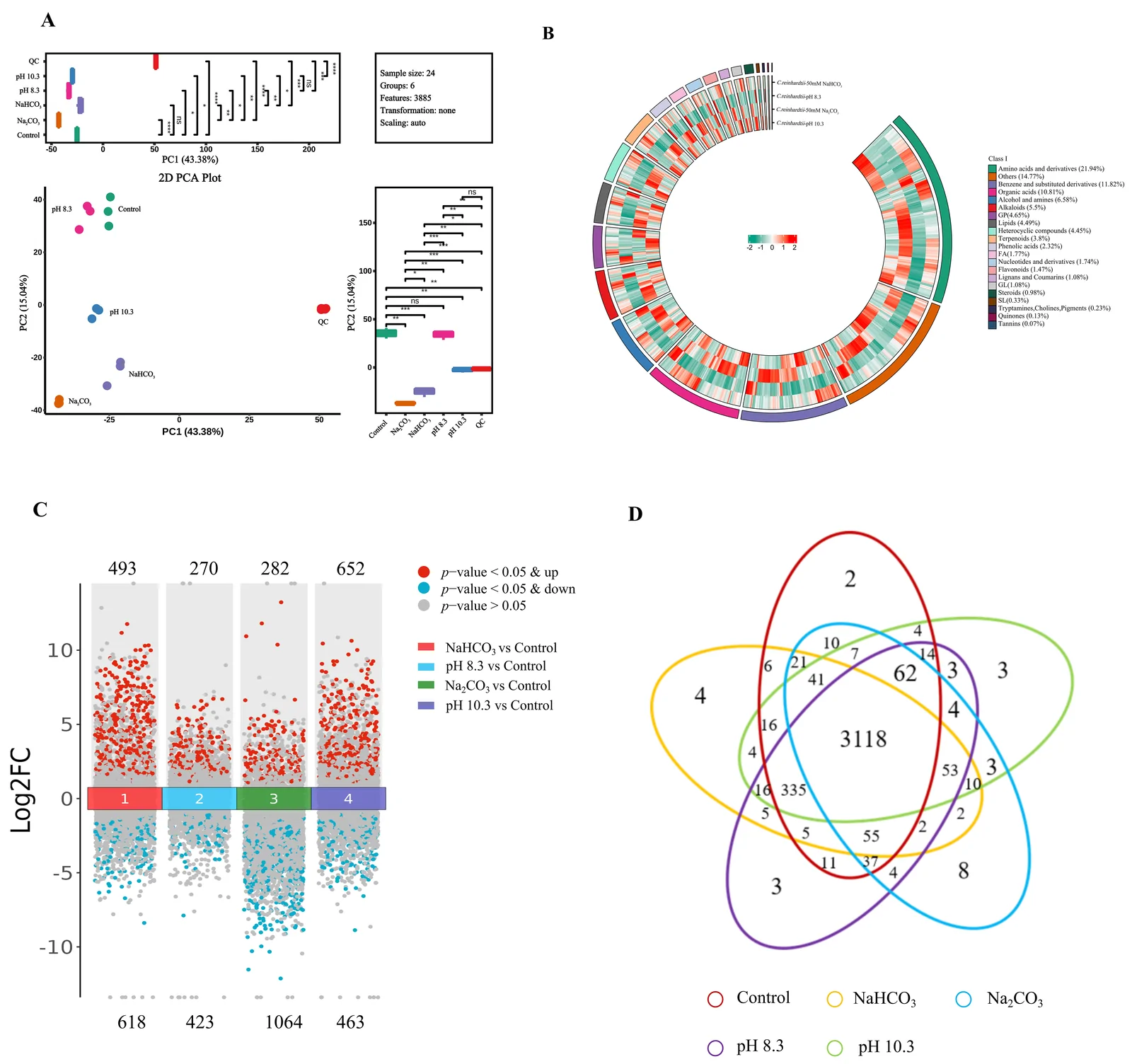
Metabolomic profile analysis of *C. reinhardtii* under alkaline stress. (**A**) The PCA. Asterisks indicates significant differences, * *p* < 0.05, ** *p* < 0.01, *** *p* < 0.001, **** *p* < 0.0001, and ns indicates no significant difference. (**B**) Circular heat map of metabolite categories. The heat map displayed the major metabolite classes and their relative abundance changes across different treatment groups. Color bars indicate relative changes in expression levels, with red representing up-regulation and green representing down-regulation. (**C**) The volcano plot of differential metabolites between NaHCO_3_ vs. Control, and Na_2_CO_3_ vs. Control, pH 8.3 vs. Control, and pH 10.3 vs. Control. Red dots represented up-regulation and blue dots represented down-regulation. (**D**) The Venn diagram shows the shared and unique metabolites with significant differences between each comparison.

In order to explore the mechanism by which *C. reinhardtii* improves soil and its role in plants in response to alkaline stress, the metabolic profile of *C. reinhardtii* in response to different alkaline stresses was explored by means of a non-targeted metabolomics analysis of the differences in metabolites (Figure 5). The PCA plot showed evident clustering-separation trends of metabolite profiles between the treatment groups and the control group, indicating that alkaline stress significantly drove metabolic rearrangements of *C. reinhardtii*. With the exception of the pH 8.3 group, which demonstrated no significant differences from the control group (Figure 5A), the remaining groups exhibited notable variations. This tendency appears to be in accordance with the preceding research findings.

The ring heatmap further illustrated the relative abundance changes in the main metabolite classes across different treatment groups (Figure 5B). Key metabolite categories, such as amino acids and derivatives and organic acids and derivatives, exhibited significant up-regulation or down-regulation trends under different alkaline conditions. These findings reflect the metabolic adaptation strategies of *C. reinhardtii* to alkaline environments.

Concurrently, the volcano plot delineated the quantitative alterations in DPMs (*p* < 0.05, |log2 fold-change| ≥ 1) in response to disparate alkaline stress treatments (Figure 5C). The results showed that 493 DPMs were found to be up-regulated and 618 down-regulated under NaHCO_3_ stress compared to the control group, while 224 DPMs were found to be up-regulated and 119 down-regulated under pH 8.3 stress. These results exhibited relatively mild changes for microalgae compared to the NaHCO_3_ group. Furthermore, 282 DPMs were up-regulated and 1064 were down-regulated under Na_2_CO_3_ stress, while 652 up-regulated and 463 down-regulated DPMs were observed under pH 10.3, suggesting a differential metabolic response mechanism to Na_2_CO_3_ and pH 10.3 in *C. reinhardtii*.

The Venn diagram reveals a significant overlap in metabolites between the treatment groups and the control group, including 3118 shared metabolites, while each treatment group also exhibits unique metabolites (Figure 5D). This finding demonstrates that *C. reinhardtii* employs both conserved metabolic response mechanisms and unique adaptive strategies under varying alkaline conditions.

These findings support the hypothesis that *C. reinhardtii* adapts to alkaline environments by regulating its metabolite composition, particularly amino acids and carbohydrates, thereby providing metabolic evidence for understanding its effects on soil improvement, regulating soil pH, or nutrient cycling through metabolites.

### 3.5. Analysis of the Metabolic Network of C. reinhardtii Under Alkaline Stress

**Figure 6:**
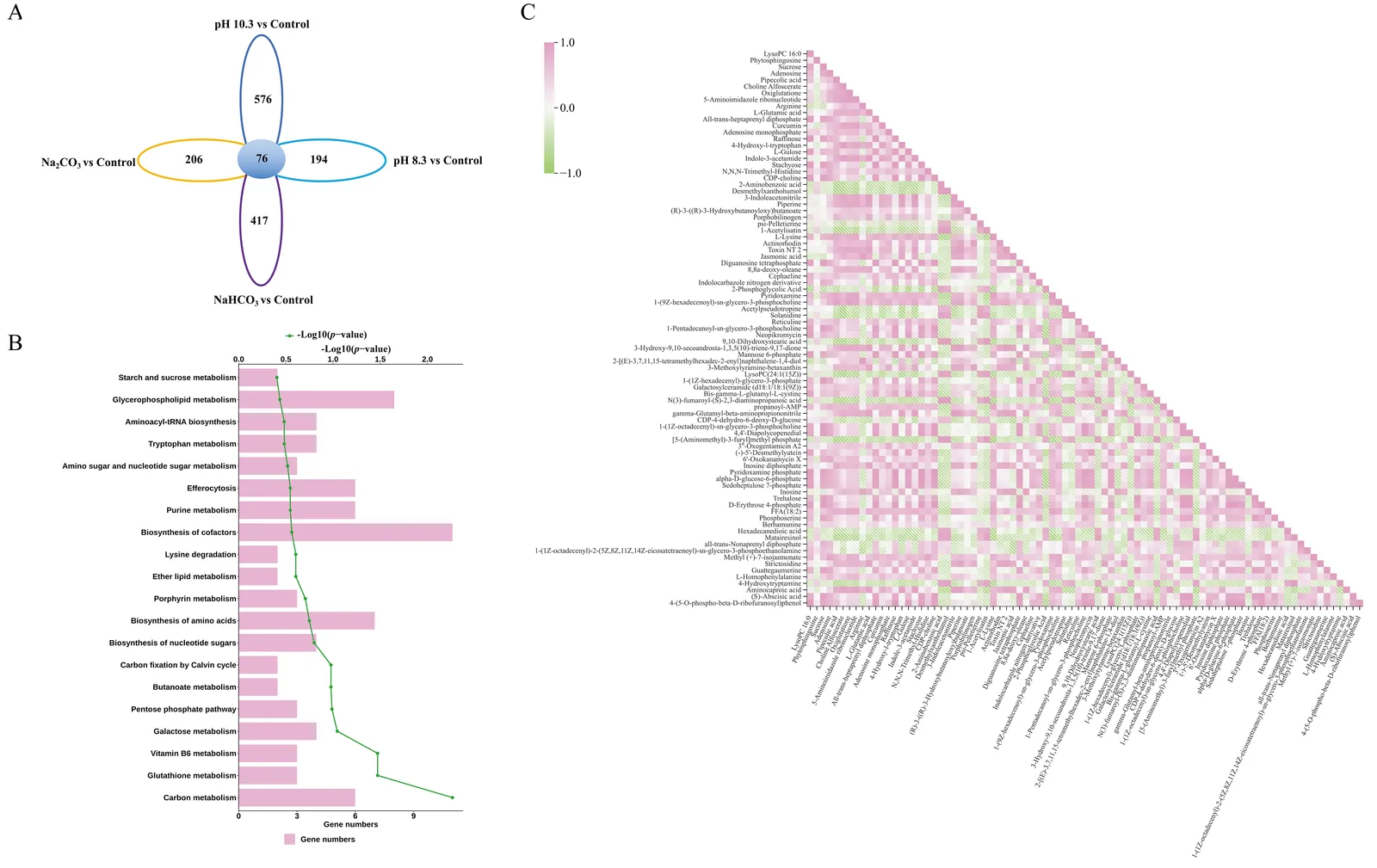
Positive response of *C. reinhardtii* to alkaline stress. (**A**) Venn diagram of up−regulated DPMs of *C. reinhardtii* under different alkaline treatments (Na_2_CO_3_ vs. control, NaHCO_3_ vs. control, pH 8.3 vs. control, pH 10.3 vs. control). (**B**) KEGG enrichment analysis of all up−regulated DPMs responding to four alkaline stresses. (**C**) Correlation analysis of DPMs enriched in KEGG pathways. Pink for high expression, green for low expression.

To elucidate the mechanisms underlying the response of *C. reinhardtii* to alkaline stress and its potential role in soil improvement, a comprehensive metabolic analysis was performed in four comparison groups: Na_2_CO_3_ vs. control, NaHCO_3_ vs. control, pH 8.3 vs. control, and pH 10.3 vs. control (Figure 6). Due to the complex composition of soda saline–alkali soils, which impose not only high pH stress, but also associated ionic stresses derived from CO_3_^2−^ and HCO_3_^−^, we screened all metabolites that were significantly up-regulated under at least one of the four alkaline treatments. Venn diagram analysis was performed using these up-regulated metabolites, followed by KEGG enrichment and correlation analyses.

Venn analysis revealed that a total of 1159 differentially produced metabolites (DPMs) accumulated significantly under at least one alkaline stress condition, among which 76 metabolites were consistently accumulated across the four treatments (Figure 6A). The results demonstrate that alkaline stressors, including elevated pH, CO_3_^2−^ and HCO_3_^−^, induce extensive reprogramming of the metabolic profiles of *C. reinhardtii*, manifesting in treatment−specific responses and shared metabolic adjustments. Furthermore, the 76 commonly accumulated DPMs are likely to represent the core metabolites responsible for the adaptation of *C. reinhardtii* to alkaline stress.

KEGG enrichment analysis was performed on the basis of up−regulated DPMs under alkaline stress, and 85 substances were identified with annotations in KEGG pathways (Figure 6B). The results of the enrichment analysis indicated that DPMs were significantly enriched in multiple fundamental metabolic pathways, including those associated with carbohydrate metabolism, such as starch and sucrose metabolism; lipid metabolism, such as glycerophospholipid metabolism; amino acid metabolism, particularly lysine degradation and tryptophan metabolism; nucleotide synthesis; and cofactor biosynthesis pathways. These results suggest that the tolerance of *C. reinhardtii* to alkaline stress may be enhanced by modulating these core metabolic pathways.

Furthermore, correlation analysis of the 85 up-regulated substances enriched in the pathway revealed that a subset of these metabolites showed significantly positive correlations under alkaline stress, while others showed markedly negative correlations. Collectively, these observations highlight sophisticated and intricate regulatory networks governing metabolic adaptation to alkaline environments (Figure 6C).

**Figure 7:**
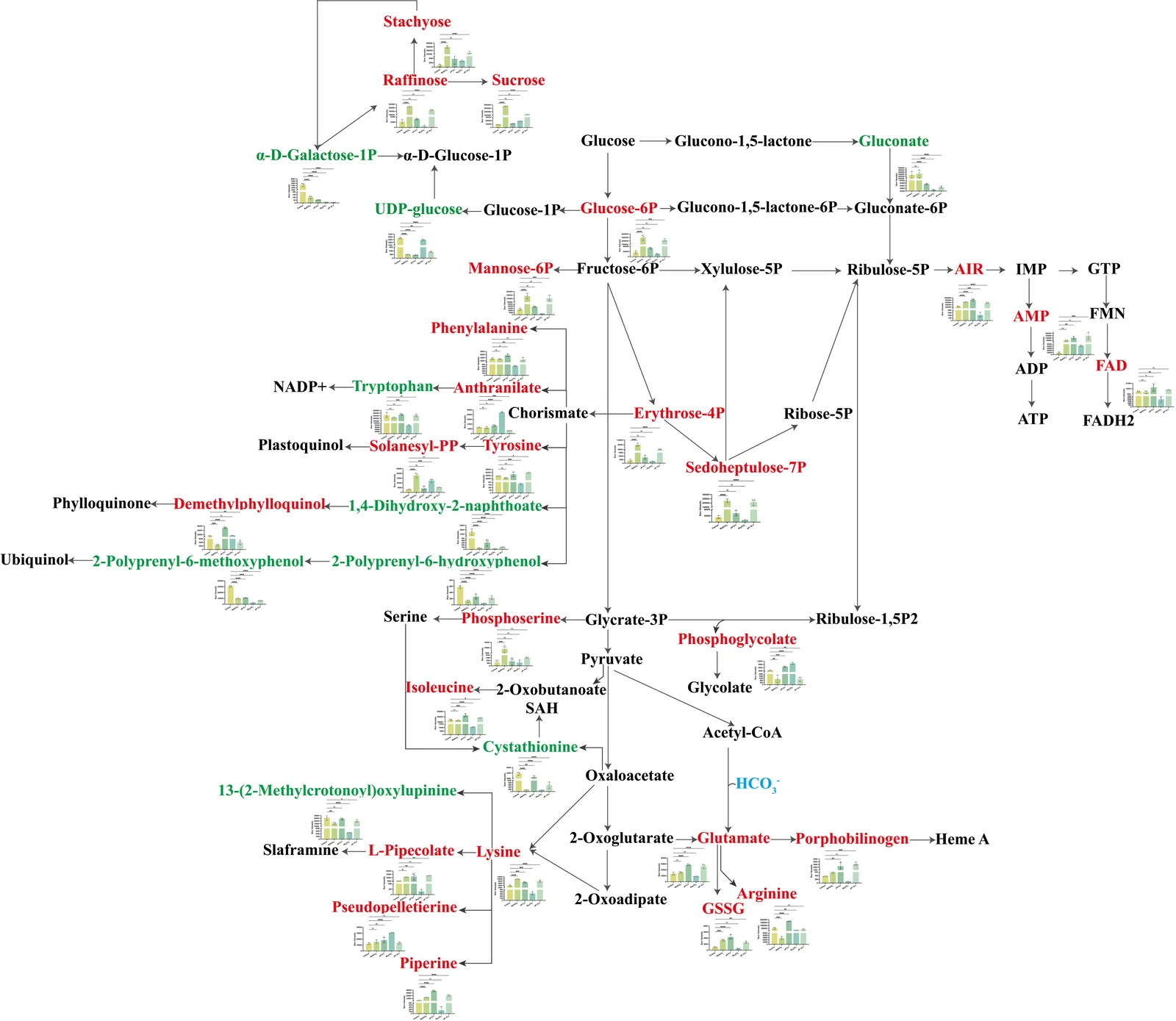
Analysis of metabolic networks in *C. reinhardtii* under alkaline stress. Black indicates undetected metabolites, red indicates detected up-regulated metabolites, and green indicates detected down-regulated metabolites. The bar chart is plotted with the raw intensity of DPMs. Asterisks indicates significant differences, * *p* < 0.05, ** *p* < 0.01, *** *p* < 0.001, **** *p* < 0.0001, and ns indicates no significant difference.

To obtain a comprehensive understanding of the metabolic regulatory landscape of *C. reinhardtii* in response to alkaline stress, an integrated metabolic pathway map was constructed based on our metabolic data obtained in this study and data from previous studies. The map provides a visual representation of the key metabolic pathways, including cofactor biosynthesis, glycerophospholipid metabolism, amino acid biosynthesis, purine metabolism, carbon metabolism, and galactose metabolism (Figure 7).

A total of six metabolites associated with the galactose metabolism pathway were identified, including stachyose, sucrose, raffinose, and glucose-6-phosphate (glucose-6P). These metabolites were found to be accumulated, particularly under conditions of NaHCO_3_ and pH 10.3, indicating that *C. reinhardtii* may employ an osmoregulatory response to alkaline stress. However, α-D-galactose-1-phosphate (α-D-galactose-1P) and uridine diphosphate-glucose (UDP-glucose) were down-regulated, hypothesizing that they may serve as precursor substances for conversion to stachyose, sucrose, and raffinose when encountered in an alkaline environment. Glucose-6P also participates in carbon metabolism. In this pathway, glucose-6P was altered to fructose-6P, following conversion to erythrose-4P and sedoheptulose-7P, which also accumulated specifically under stress from NaHCO_3_ and pH 10.3. In the context of the carbon metabolism pathway, glutamate accumulation was observed under high pH conditions, just in the pH 8.3 and 10.3 groups. The relationship between the amino acid biosynthesis pathway and the carbon metabolism pathway is such that they intersect. Chorismate, a downstream metabolite of erythrose-4P in carbon metabolism, has been shown to serve as a central hub for the synthesis of phenylalanine, anthranilate, solanoyl-PP, and demethylphytoquinol via the biosynthesis of amino acids under alkaline stress. Furthermore, glycate-3P was used to accumulate phosphoserine and isoleucine in this pathway. In the amino acid biosynthesis pathway, oxaloacetate and 2-oxoadipate function as precursors for lysine, which serves as a metabolic hub to convert further to L-pipercolate, pseudopelletierine, and piperine. It is noteworthy that glutamate also participates in this metabolic process, ultimately transforming into arginine. Furthermore, the cofactor biosynthesis pathway was found to be closely associated with amino acid biosynthesis and the carbon metabolism pathway. In this pathway, AIR, AMP, and FAD were found to be up-regulated. The purpose of this up-regulation is to ensure that the cell maintains energy balance by regulating the level of nucleotide metabolism. Furthermore, the accumulation of GSSG was observed, indicating intracellular redox imbalance in *C. reinhardtii*. In addition, the metabolites associated with the plastoquinol and phylloquinone biosynthesis pathway were detected in *C. reinhardtii* under alkaline stress conditions, resulting from the accumulation of solanoyl-PP and demethylphylloquinol. This phenomenon mirrors the impact of alkaline stress on the photosynthetic electron transport chain.

These results support the mechanism whereby *C. reinhardtii* executes a coordinated response to alkaline stress through the regulation of carbon metabolism, amino acid, and cofactor biosynthesis pathways. The accumulation of differential metabolites mirrors its adaptation to alkaline environments and provides metabolic evidence to decipher its potential function in soil improvement.

### 3.6. Correlation Analysis Between Stress Factors and Annotated Metabolites of the KEGG Pathway

**Figure 8:**
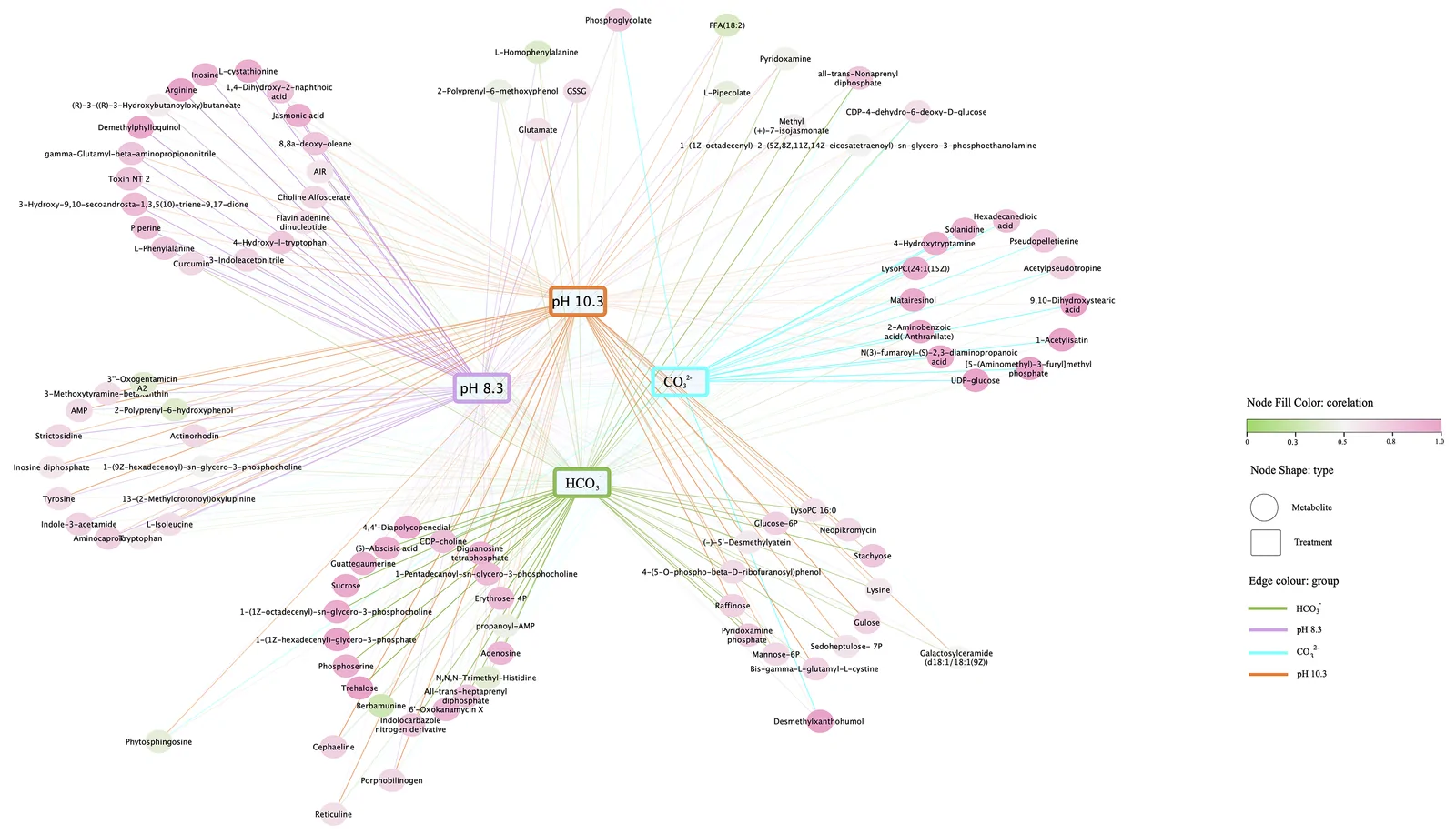
Correlation network analysis between stress factors (NaHCO_3_, pH 8.3, Na_2_CO_3_, pH 10.3) and KEGG pathway−annotated metabolites. Edge color indicates the corresponding treatment group; node color of metabolites represents the value of the correlation coefficient with the related treatment, with pink indicating stronger correlation and green indicating weaker correlation. Nodes were clustered according to their closely associated treatments to visualize the module structure of treatment−metabolite correlations.

The objective of this was to systematically dissect the association patterns between alkaline stress factors and up-regulated metabolites annotated on the KEGG pathway. To this end, a correlation network was constructed using Cytoscape software, with a focus on four alkaline conditions: NaHCO_3_, pH 8.3, Na_2_CO_3_, pH 10.3 (Figure 8).

From the perspective of the distribution of the associated modules, different stress factors correspond to specific modules of metabolic response. Treatment with pH 10.3 was associated with the highest number of metabolites, 55 in total, and the correlation ranged from 0.19 to 0.719, indicating a stronger correlation between high alkaline stress and most annotated metabolites. The metabolites implicated included reticuline, 3-methoxytyramine-betaxanthin, tyrosine, amp, porphobilinogen, neopikromycin, L-gulose, bis-gamma-L-glutamyl-L-cystine, cephaeline, and so on. These covered multiple pathways such as amino acid metabolism and cofactor biosynthesis. This suggests that strong alkaline environments significantly shape the overall metabolic network of *C. reinhardtii*. The correlation indicates that Na_2_CO_3_ stress, which has a pH of 10.3, exhibits a strong correlation with 19 metabolites, including CDP-4-dehydro-6-deoxy-D-glucose, acetylpseudotropine, pseudopelletierine, phosphoglycolate, hexadecanedioic acid, phytosphingosine, solanidine, 4-hydroxytryptamine, lysoPC (24:1(15Z)), etc. Among them, the correlation of matairesinol, anthranilate, UDP-glucose, 1-acetylisatin, and 9,10-dihydroxystearic acid was greater than 0.95. CO_3_^2−^ is one of the stress factors in the soda–saline soil, dissociated from Na_2_CO_3_, and the corresponding metabolic module may be the same as the stress of Na_2_CO_3_. This indicates that CO_3_^2−^, as one of the core ions of alkaline stress, primarily regulates carbohydrate metabolism and lipid metabolism pathways in *C. reinhardtii* when responding to carbonate-induced alkaline stress. More importantly, the substances mapped by stress at Na_2_CO_3_ and high pH are quite different, indicating the different response mechanisms of microalgae.

Treatment with pH 8.3, a weakly alkaline environment, corresponded to the third largest metabolic module, with 45 associated metabolites such as 1,4-Dihydroxy-2-naphthoic acid, piperine, jasmonic acid, L-cystathionine, demethylphylloquinol, and arginine, etc., mainly concentrated in biosynthesis of terpenoid-quinone, alkaloid metabolism, lipid metabolism, amino acid metabolism, and vitamin biosynthesis. The stress of NaHCO_3_, at pH 8.3, occupied the second-largest module, including guattegaumerine, (S)-abscisic acid, phosphoserine, 1-(1Z-octadecenyl)-sn-glycero-3-phosphocholine, 4,4′-diapolycopenedial, sucrose, 1-(1Z-hexadecenyl)-glycero-3-phosphate, trehalose, etc. HCO_3_^-^ dissociated from NaHCO_3_, which is also the main component of soda soil, is primarily associated with the metabolism of amino acids and carbohydrates.

The results indicate that *C. reinhardtii* exhibits different metabolic responses to different types of alkaline stress. This analysis elucidates the core metabolic modules of *C. reinhardtii* in soda soil, a complex system that includes various alkaline stress factors. Furthermore, *C. reinhardtii* in soda saline soils may activate distinct metabolic modules to adapt to varying degrees of alkalinity, thus contributing to the improvement of the physicochemical properties of the soil through the release or transformation of metabolites. The findings provide critical information on the metabolic regulatory mechanisms underlying alkaline adaptation and offer a foundation for targeted metabolic regulation to improve alkaline soils using microalgae.

### 3.7. Correlation Analysis of Annotated Metabolites of the KEGG Pathway and Up-Regulated Metabolites Under Alkaline Conditions

**Figure 9:**
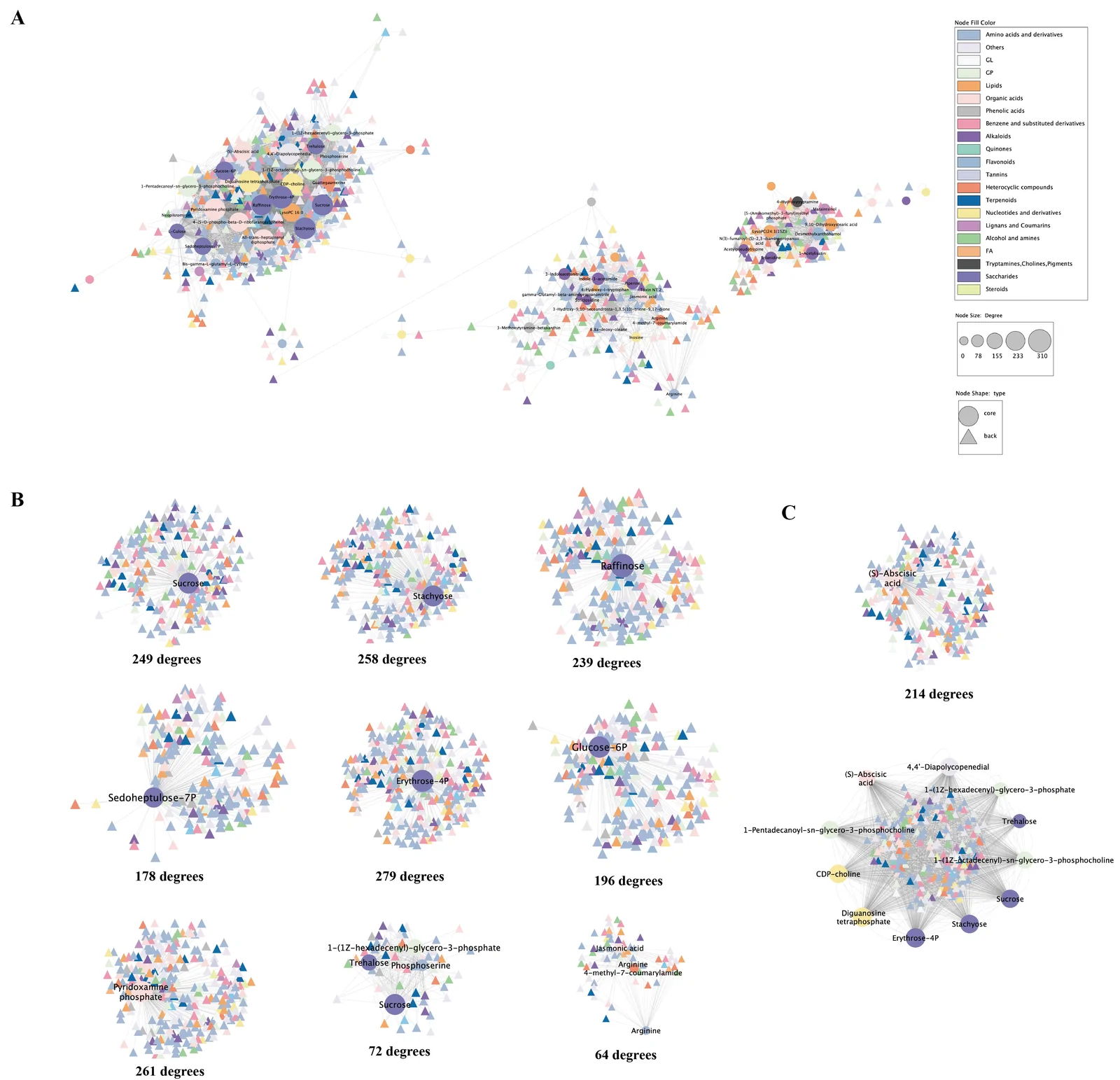
Correlation network of KEGG pathway−annotated metabolites and up−regulated metabolites under any of the alkaline conditions with a correlation coefficient > 0.9. (**A**) Global correlation network. Node size is proportional to the degree of connectivity, with larger nodes representing hub metabolites with higher connectivity. Nodes are distinguished by shape: core metabolites and background metabolites are represented by different shapes. Core metabolites are those with a correlation coefficient > 0.5 between KEGG pathway-annotated metabolites and treatments. Background metabolites are up−regulated metabolites under any of the alkaline conditions. (**B**) Magnified view of selected key nodes in (**A**), highlighting critical hub metabolites and their local connections. (**C**) ABA nodes’ magnified view and connections with saccharides.

In order to ascertain whether the biotransformation of metabolites annotated on the KEGG pathway in *C. reinhardtii* under alkali stress is coordinated with other metabolites that are up-regulated, we defined up-regulated compounds with available KEGG pathway annotations and a correlation coefficient > 0.5 against stress factors (data analyzed from Figure 8) as core metabolites, while all up-regulated metabolites to alkaline stress were used as background metabolites. A correlation analysis was then performed between the aforementioned annotated metabolites from the KEGG pathway and the metabolites that were found to be up-regulated. This analysis was performed using the software program Cytoscape, with the aim of identifying additional metabolites that were responsive to alkali stress (Figure 9).

The global correlation network of KEGG pathway-annotated metabolites and up-regulated metabolites under any of the alkaline conditions with a correlation coefficient > 0.9 was shown in Figure 9A, in which the size of the node is proportional to the degree of connectivity. The core and background metabolites with connectivity degree <10 were shown. The core and background metabolites were distinguished by different shapes, intuitively displaying the interaction network between metabolites. We identified saccharides, organic acids, GP, and nucleotides and derivatives as the largest network, indicating their close association with metabolic processes during *C. reinhardtii*’s response to alkaline stress. The second largest network comprised alkaloids, phenolic acids, and other compounds, reflecting their significant interactions. The interactions between the core and background substances were extensive and complex. In order to conduct a more thorough analysis of the role played by background substances in the biotransformation of core substances under alkaline stress, the networks of several key core substances with high connectivity were subjected to magnification (Figure 9B).

As illustrated in Figure 9B, the key hub nodes, including sucrose, stachyose, and raffinose, exhibit high levels of connectivity, with degrees of 249, 258, and 239, respectively. These sugars have been identified as central hub molecules in response to alkali stress, mediating extensive metabolic crosstalk involving amino acids, lipids, organic acids, and other classes of metabolites. This facilitates carbon reallocation and energy metabolic reprogramming. Glucose-6-phosphate (glucose-6P), erythrose-4-phosphate (erythrose-4P), and sedoheptulose-7-phosphate (sedoheptulose-7P), which are also classified as saccharides, exhibited a significant propensity to form connections with back metabolites. Pyridoxamine phosphate is another key node of the hub with 261 degrees, constituting one of the active forms of vitamin B6 and playing a crucial role in amino acid metabolism. In addition, phosphoserine, an amino acid derivative formed by phosphorylation of serine, was associated closely with sucrose and is critical for the regulation of cellular signal transduction and protein function. Jasmonic acid, a plant hormone involved in the regulation of plant growth, development, and defense responses, also shows a close association with arginine [35]. In particular, another plant hormone, namely ABA, showed direct correlations with sucrose, stachyose, trehalose, and other saccharides, with a connectivity degree of 214 (Figure 9C). These results indicate that the ABA signaling pathway and the carbohydrate metabolism pathway are tightly interconnected, forming a key regulatory module for *C. reinhardtii* to adapt to alkaline environments by coordinating osmotic adjustment and energy metabolism to maintain cell homeostasis.

Collectively, correlation network analysis demonstrates that the metabolic network of *C. reinhardtii* under alkaline stress exhibited a highly connected architecture centered on saccharide metabolites. The present study investigates the function of ABA as a critical regulator that synergistically responds to alkali stress by modulating sugar metabolism pathways. These findings provide important information on the alkali-tolerant mechanism of *C. reinhardtii* and its metabolic regulation during soil remediation.

## 4. Discussion

### 4.1. Key Metabolic Adaptation of C. reinhardtii to Alkaline Stress

To comprehensively understand the regulatory mechanism of metabolites of *C. reinhardtii* under alkaline stress, we integrated the main metabolic pathways based on a literature review and untargeted metabolomics data (Figure 7). These pathways reveal the core metabolic strategies of *C. reinhardtii* in response to high pH and carbonate stress, mainly including cofactor biosynthesis, glycerophospholipid biosynthesis, amino acid biosynthesis, purine metabolism, carbon metabolism, tropane, piperidine and pyridine alkaloid biosynthesis, as well as galactose metabolism.

It is clear that carbon metabolism pathways are central to the alkaline adaptation of *C. reinhardtii*. It was observed that glucose-6P underwent a change in carbon metabolism in comparison with the control. The initial conversion of the substrate to fructose-6-phosphate (fructose-6P), subsequently resulted in its flow into the sedoheptulose-7P and erythrose-4P pathways. In particular, the levels of erythrose-4P and sedoheptulose-7P increased significantly in response to the application of NaHCO_3_ and pH 10.3. Sedoheptulose-7, a kind of sugar phosphate and a component of the reductive pentose phosphate cycle, performs a pivotal function within the metabolic network of carbon rearrangement [36]. Erythrose-4P is the common starting point in the biosynthesis of the three aromatic amino acids, including l-Phenylalanine, L-tyrosine, and L-tryptophan [37]. The present study demonstrated that erythrose-4P was converted to tyrosine and phenylalanine under alkaline conditions. Such redistribution of carbon skeletons may contribute to the maintenance of intracellular osmotic balance and the mitigation of excessive dehydration under high pH stress. Furthermore, amino acid metabolism may be downstream of the carbon metabolic pathway, while erythrose-4P is merely the starting precursor to amino acid synthesis and is a potential hub connecting carbon metabolism and amino acid metabolism. The results indicated that glutamate was accumulated under high pH conditions, a phenomenon that may be associated with alterations in carbon metabolic pathways. Furthermore, glutamate, a multifunctional amino acid present in plants, has been shown to directly influence the relationship between nitrogen and carbon metabolism [38]. Furthermore, the potential of glutamate to engage in amino acid synthesis has been demonstrated, with a subsequent conversion to arginine under alkaline stresses observed in *C. reinhardtii* within the present study. It has been hypothesized that arginine catabolism may serve a role in the mobilization of nitrogen stores, as well as the fine-tuning of developmental and defensive mechanisms in response to stress [39]. These data suggested that *C. reinhardtii* could effectively cope with alkaline stressors by adjusting carbon flow, carbohydrate and amino acid accumulation. It is notable that the galactose metabolic pathway is activated in *C. reinhardtii* in response to alkaline stress. A total of six metabolites related to this pathway were identified, including stachyose, sucrose, raffinose, and glucose-6P, which have been shown to accumulate to a significant extent under the conditions of NaHCO_3_ and pH 10.3. Raffinose is a polysaccharide comprising D-galactose, and it is present in a wide range of higher plants [40]. In the present study, the presence of the aforementioned compound was also detected in *C. reinhardtii* in an alkaline environment. It has been established that the synthesis of raffinose and stachyose is catalyzed by raffinose synthase and stachyose synthase, respectively, with sucrose and galactinol as the substrates [40]. Raffinose is hydrolyzed by α-galactosidase to produce sucrose and galactose. However, an increasing level of stachyose, sucrose, and raffinose was observed in this study. The aim of this observation was to activate the corresponding enzymes in this pathway under alkaline stress. This suggests that they may act as carbohydrate reserves in *C. reinhardtii*, helping in tolerance to abiotic stress [41]. It is noteworthy that the levels of α-D-galactose-1P and UDP-glucose were found to be down-regulated. According to the KEGG database, α-D-galactose-1P serves as a precursor for UDP-galactose, while UDP-glucose is the predominant precursor for the synthesis of carbohydrates. Collectively, these findings suggest that carbon sources may be redistributed to metabolic pathways that are more favorable for plant survival and tolerance under alkaline stress, thereby leading to a decrease in the accumulation of UDP-glucose and its precursor, α-D-galactose-1P.

Furthermore, the metabolic pathways associated with amino acids were also found to be subject to alteration under alkaline conditions. A significant accumulation of amino acids was observed. Concurrently, amino acids such as tyrosine, phosphoserine, lysine, and glutamate accumulated and functioned as precursors that were subsequently entered into the other pathways, suggesting that the amino acid metabolic network underwent a process of reprogramming. In particular, the accumulation of lysine, which functions as a metabolic hub. This is further converted into nitrogen-containing compounds, including L-pipercolate, pseudopelletierine, and piperine. These amino acids and nitrogen-containing derivatives have been shown to participate in protein synthesis and cell structure construction, and they may also play a role in *C. reinhardtii* stress responses. Kawade (2023) further illustrated that plants incorporate acquired carbon and nitrogen into amino acid metabolism, thereby enabling the production of building blocks of proteins and precursors of various metabolites [42]. Amino acids such as arginine were regarded as functional amino acids [42]. In addition, Wu (2023) reported that microalgae are a valuable source of amino acids [43]. Concurrent studies also suggested that amino acids not only play a pivotal role in plant growth and development, but also function as an energy source capable of replacing sugars as electron donors to the mitochondrial electron transport chain [44]. Furthermore, microalgae have the potential to supply amino acids that have been demonstrated to augment plant defense responses [45].

The cofactor biosynthesis pathways have been shown to be closely related to amino acid metabolism and carbon metabolism. Cofactors, such as 5-aminoimidazole ribonucleotide (AIR), adenosine monophosphate (AMP) and flavin adenine dinucleotide (FAD), were found to be up-regulated in *C. reinhardtii* under alkaline stress. Cofactors are essential for the efficiency of enzymatic reactions, and it is imperative to maintain cofactor balance to ensure the effective functioning of core biochemical processes [46,47]. FAD, a flavin cofactor, is indispensable for plant metabolism, supporting processes such as photosynthesis, photorespiration, mitochondrial electron transport, nitrogen assimilation, and cellular redox balance. This indicates that it performs a similar function in microalgae [46]. Furthermore, the accumulation of oxidized glutathione (GSSG) clearly indicates that alkaline stress induces an oxidative stress response [48]. In order to combat the effects of this oxidative stress, cells initiate antioxidant defense systems. Meanwhile, the modules of plastoquinol and phylloquinone biosynthesis have been activated, reflecting the impact of alkaline stress on the photosynthetic electron transport chain and the efforts to protect photosynthetic organs from damage [49,50,51,52]. In addition, molybdenum cofactor (Moco) serves as an essential prosthetic group for five conserved molybdoenzymes present in *C. reinhardtii*, including nitrate reductase (NR), sulfite oxidase (SO), xanthine dehydrogenase (XDH), aldehyde oxidase (AO), and mitochondrial amidoxime-reducing component (mARC) [53]. In our metabolomic dataset, we did not observe significant enrichment of the Moco biosynthesis pathway under alkaline stress. It should be emphasized that untargeted metabolomics mainly reflects changes in small-molecule intermediates. Moco-related homeostasis can also be regulated at transcriptional, post-transcriptional or post-translational levels.

Notably, *Chlamydomonas sp*. grows purely photoautotrophically and requires ammonia as a nitrogen source because its nuclear genome lacks some of the genes required for nitrogen metabolism [54]. This feature potentially highlights the critical impact of nitrogen availability on *Chlamydomonas sp*. Previous studies have demonstrated that *C. reinhardtii* could utilize a wide range of nitrogen substrates, and nitrogen forms substantially affect physiology and metabolic profiles of *C. reinhardtii*. For example, Franco (1996) showed that *C. reinhardtii* was able to grow with the ammonium salt of l-phosphinothricin (PPT), the main active component of the herbicide, supported by an l-amino acid oxidase located at the periplasm [55]. Urea as a nitrogen source could be used by *C. reinhardtii* to promote changes in carbohydrate and amino acid metabolism, as well as in lipid production and fatty acid profile [56]. Rosa (2023) reported that ammonium and urea could lead to increased cell growth and a higher accumulation of lipids, while urea stimulates growth in terms of cell number and dry weight and higher photosynthesis was verified in the late logarithmic phase compared to ammonium [57]. Yasar (2023) reported that nitrate was the preferred form of nitrogen source under heterotrophic conditions, while nitrogen sources did not present any significant influences in the phototrophic cultivation [58]. All experiments in the present study were carried out in TAP medium with ammonium as the nitrogen source. However, different nitrogen sources may lead to divergent stress phenotypes. Further investigations are required to compare the alkaline tolerance of this isolate under different nitrogen source supplies.

### 4.2. Potential Roles of C. reinhardtii Metabolic Substances in Soda Soil Amelioration and Plant Growth

This study systematically investigated the physiological responses and metabolic adaptation mechanisms of *C. reinhardtii* under alkaline stress, as well as its potential application in the remediation of saline–alkali soil. In order to cope with alkaline stress, *C. reinhardtii* initiates a broad range of metabolic adaptation strategies. The results of this study demonstrated that this microalga not only exhibits differential tolerance to various alkaline stress conditions (including NaHCO_3_, Na_2_CO_3_, pH 8.3, and pH 10.3), but also accumulates numerous key metabolites through complex metabolic reprogramming.

Additionally, a systematic analysis of the relationship between stress factors and microalgal metabolites is conducted. Sucrose, stachyose, raffinose, glutamate, arginine, and antioxidant substances such as glutathione disulfide have been shown to participate in soil improvement through multiple pathways. In particular, *C. reinhardtii* could effectively reduce the pH value, EC, and the concentration of HCO_3_^−^ and CO_3_^2−^ in soda saline–alkali soil. This finding suggests that *C. reinhardtii* could offer a viable approach for the utilization of saline–alkali soil. Previous studies reported that trebouxiophyte strains screened and identified from Songnen Plain showed extreme tolerance to high concentrations of NaHCO_3_, but the application to plant seedlings remained a limitation [59]. Youssef (2022) reported that *Arthrospira platensis*, *Chlorella vulgaris*, *Nostoc muscorum*, and *Anabaena azollae* promote plant growth and improve soil microbial activity, which in turn leads to a reduction in soil pH [60].

These findings collectively reveal the significant potential of *C. reinhardtii* and its metabolites in the ecological restoration of saline–alkali lands, which is consistent with recent studies reporting the positive role of microalgae in improving soil quality. For instance, Zhou (2023) reported that Azotobacter beijerinckii inoculation treatment combined with live *Chlorella pyrenoidosa* effectively reduced soil pH and improved soil phosphorus availability [23]. In a similar vein, Zhang (2024) posited that microalgal inoculation contributes to the remediation of saline soils by increasing soil organic matter storage [61].

To further clarify the underlying mechanisms by which *C. reinhardtii* improves alkaline soil, several key pathways associated with its metabolites were systematically discussed. First, nitrogen-containing compounds such as glutamate and arginine accumulated by *C. reinhardtii* not only serve as osmoregulators for the microalga under alkaline stress but also participate in soil nutrient cycling, improving soil nitrogen availability, and further facilitating soil improvement [62].

Furthermore, sugars, such as sucrose, stachyose, and raffinose, produced by *C. reinhardtii,* may play a crucial role in promoting photosynthetic efficiency. *C. reinhardtii* has a well-characterized inorganic carbon-concentrating mechanism (CCM), which enables efficient utilization of HCO_3_^−^ in the soil as a carbon source for photosynthesis [63]. Consistent with this, recent studies have confirmed that the NAR1.1 and NAR1.5 transporters located on the chloroplast membrane of *C. reinhardtii* exhibit significant HCO_3_^−^ transport activity, which is essential for the efficient operation of CCM [64,65].

Additionally, *C. reinhardtii* can indirectly improve soil structure and fertility by regulating soil microecology through its metabolites. Specifically, the metabolic activities of *C. reinhardtii* promote the succession and diversity of soil microbial communities, establishing a beneficial microecological balance [60,66,67]. These soil microorganisms also participate in organic matter decomposition, nitrogen and phosphorus cycling, and the enhancement of soil enzyme activity, thus improving soil physical and chemical properties [68].

Collectively, these findings provide an important scientific basis and technical support for the application of *C. reinhardtii* in the ecological restoration of saline–alkali land and plants’ responses to alkaline stress. However, the present experiment did not include a TAP medium control group. Even though Hoagland nutrient solution may be more suitable for rice seedlings than TAP medium, TAP medium also contains soluble bioactive metabolites that may contribute to plant growth. Further experiments should be designed to clarify whether the observed plant growth−promoting effects are attributable to *C. reinhardtii* itself or to the soluble bioactive metabolites present in the TAP medium. In addition, future research should further explore the mechanisms of interaction between *C. reinhardtii* metabolites and soil microecology, as well as its long−term ecological effects in larger-scale and more complex saline–alkali environments, laying the foundation for the widespread application of *C. reinhardtii* in sustainable agriculture and ecological management.

### 4.3. Metabolic Differences Among NaHCO_3_, Na_2_CO_3_, pH 8.3, pH 10.3 Stresses

Despite the pH of NaHCO_3_ being equivalent to that of the pH 8.3 treatments, the physiological stimulation of NaHCO_3_ treatment in *C. reinhardtii* is more significant. This suggests that, in addition to pH, ions have a profound impact on microalgal metabolic adaptation and physiological responses. NaHCO_3_ may induce a high pH in the environment; furthermore, of greater significance is its ability to introduce HCO_3_^−^ ions. HCO_3_^−^ is an important inorganic carbon source for *C. reinhardtii* photosynthesis, especially in high pH environments where the solubility of CO_2_ is known to decrease, thus making HCO_3_^−^ the main carbon source [65]. Consequently, the administration of NaHCO_3_ has been demonstrated to augment the physiological activity of *C. reinhardtii*. In addition, non-targeted metabolomics analysis revealed significant differences in metabolites of *C. reinhardtii* under NaHCO_3_ and pH 8.3 treatments, which may reflect their specific physiological strategies to adapt to different stresses. Firstly, some metabolites were accumulated under NaHCO_3_ stress, including trehalose, sucrose, 4,4′-diapolycopenedial, phosphoserine, (S)-abscisic acid, and guattegaumerine. These metabolites have been shown to interact closely with HCO_3_^−^. Additionally, abscisic acid (ABA) has been identified as a pivotal plant hormone that plays a critical role in the regulation of osmotic stress and abiotic stress responses [69]. Although the mechanism of action of ABA in microalgae remains to be fully elucidated, studies have demonstrated a close relationship between the ABA signaling pathway and sugar signaling in plants [70]. In particular, the present study has demonstrated that ABA functions as a central regulatory hub, exhibiting a high degree of correlation with saccharides, including trehalose, sucrose, and stachyose under NaHCO_3_ stress in *C. reinhardtii*. This suggests that the accumulation of saccharides in *C. reinhardtii* may be linked to the ABA signaling pathway, thus assisting cells in coping with osmotic stress.

However, it should be noted that both Na_2_CO_3_ and pH 10.3 stress occur within a high pH environment. It can therefore be hypothesized that Na_2_CO_3_ may act as a significant inhibitor, while pH 10.3 may act as a promoter in the growth and metabolic activities of *C. reinhardtii*. The observed difference also reveals the unique and complex impact of CO_3_^2−^ on the metabolic network of *C. reinhardtii*. This discrepancy may be attributed to the direct impact of CO_3_^2−^ ions on cell structure and function, as well as their potential inhibition of the regulation of ion transport, binding, metabolism, and phenylpropanoid and flavonoid biosynthesis pathways [71,72,73]. However, such evidence has been extensively documented in plants, while studies focusing on the effects of carbonates on microalgae and their adaptive mechanisms to Na_2_CO_3_ stress remain relatively limited. This funding indicates current research gaps in the application of microalgae for the remediation of alkaline soil.

## 5. Conclusions

In this study, we mainly explored the growth and physiological responses, the metabolic adaptation mechanism, and the potential application to the improvement of *C. reinhardtii soil* under different alkaline stress factors. Firstly, *C. reinhardtii* exhibited a distinct adaptive response to varying alkaline conditions, especially demonstrating a strong tolerance under NaHCO_3_, pH 8.3 and pH 10.3 compared to the control group. On the contrary, Na_2_CO_3_ stress potentially inhibited microalgae growth and chlorophyll synthesis even at pH 10.3, showing a distinct performance. A further study showed that *C. reinhardtii* showed significant improvement effects in saline–alkali soil. With an increase in the concentration of *C. reinhardtii* and a longer cultivation duration, the pH, EC, and content of HCO_3_^−^, and CO_3_^2−^ in saline–alkali soil were significantly reduced, confirming its potential to alleviate soil alkaline stress. Moreover, *C. reinhardtii* has been shown to play a key role in rice under alkaline stress. Furthermore, nontargeted metabolomics analysis revealed the complex metabolic remodeling process of *C. reinhardtii* under alkaline stress. Metabolic network analysis further revealed the critical roles of core metabolic pathways, including carbon metabolism, amino acid metabolism, cofactor biosynthesis, etc., in adaptation to alkaline environments. Meanwhile, regulatory osmotic substances such as sucrose, raffinose, stachyose, glucose-6P, sedoheptulose-7P, and erythrose-4P and hormones such as ABA, and JA were accumulated in *C. reinhardtii* under alkaline stress as coordinating substances. Finally, although NaHCO_3_ and pH 8.3 exhibited similar pH values, they induced distinct metabolic changes, particularly unique metabolites and core response pathways. Similarly, metabolic responses to Na_2_CO_3_ and pH 10.3 showed significant differences, reflecting the specific mechanisms by which *C. reinhardtii* regulates its metabolic network to adapt to environmental conditions under different alkaline ions (CO_3_^2−^ vs. HCO_3_^−^) or varying intensities of alkaline stress. In conclusion, *C. reinhardtii* exhibits remarkable adaptability to alkaline environments, and its metabolic mechanisms are complex and closely related to stress types. These metabolic regulations not only elucidate the molecular basis of its tolerance to alkali, but also provide crucial metabolic evidence to understand how it improves saline–alkali soils by secreting metabolites such as saccharides, organic acids, amino acids, or hormones.

This study provides valuable information for understanding and using *C. reinhardtii* in improving soda soils, but there are still some limitations that need to be further studied. It is necessary to analyze related enzymatic reactions and gene expression regulation to elucidate their specific molecular mechanisms of *C. reinhardtii* in response to alkaline stress. Furthermore, our study elucidates the metabolic changes in *C. reinhardtii* under alkaline stress, but its efficacy in soil remediation depends largely on whether these metabolites can be efficiently secreted into the extracellular environment, as well as their specific effects on soil microbial communities, enzyme activities, nutrient cycling, and the pH of soil. Consequently, further experimental validation is warranted. Furthermore, it is also necessary to screen or enhance the alkaline tolerance of *C. reinhardtii* through genetic engineering or traditional breeding methods, thus improving its adaptability and beneficial effects in extreme alkaline environments. In addition, the degradation of soda–saline soil is frequently precipitated by multiple factors, including salinity, drought, alkalinity, and the presence of heavy metals. It is recommended that future research efforts concentrate on investigating the response mechanisms of *C. reinhardtii* when subjected to combined stress conditions. Additionally, further study is necessary to ascertain the universality and limitations of its application in enhancing various types of saline–alkali soils.

## Data Availability

Data will be made available on request.

## Abbreviations

Food and Agriculture Organization of the United Nations, FAO; glucose-6-phosphate, glucose-6P; glycerol-3-phosphate dehydrogenase, GPDH; lysophosphatidic acid acyltransferase, LPAAT; diacylglycerol acyltransferase, DAGAT; saturated fatty acids, SFAs; α-D-galactose-1-phosphate, α-D-galactose-1P; uridine diphosphate-glucose, UDP-glucose; 5-aminoimidazole ribonucleotide, AIR; adenosine monophosphate, AMP; flavin adenine dinucleotide, FAD; oxidized glutathione, GSSG; carbon-concentrating mechanism, CCM; erythrose-4-phosphate, erythrose-4P; sedoheptulose-7-phosphatem, sedoheptulose-7P; Principal component analysis, PCA; hierarchical cluster, HCA; kyoto encyclopedia of genes and genomes, KEGG; variable importance in projection, VIP; glutathione S-transferase, GST; catalase, CAT; Tris–acetate–phosphate, TAP; control, CK.
